# Delayed Descending Thoracic Aortic Laceration Caused by a Posterior Rib Fracture Fragment: A Case Report

**DOI:** 10.70352/scrj.cr.26-0197

**Published:** 2026-08-13

**Authors:** Makoto Yoden, Daigo Ishihara, Kohei Shibata, Keiko Ueda, Takuya Shiratori, Yoko Kataoka, Yo Kawaguchi, Jun Hanaoka, Fumihiro Shoji

**Affiliations:** Department of Thoracic Surgery, Shiga University of Medical Science, Otsu, Shiga, Japan

**Keywords:** case report, rib fractures, aortic rupture, hemothorax, thoracotomy

## Abstract

**INTRODUCTION:**

Delayed descending thoracic aortic laceration caused by a rib fracture fragment after blunt chest trauma is rare but potentially fatal. Because initial findings may appear limited, diagnosis and definitive treatment can be delayed. We report a case in which rapid progression to massive hemothorax and traumatic cardiac arrest was successfully managed by emergency thoracotomy followed by definitive aortic repair.

**CASE PRESENTATION:**

A 78-year-old woman sustained extensive bilateral rib fractures after striking her chest against a tree. She was initially managed conservatively because only a minimal hemothorax was present. Early CT showed a medially displaced posterior left 11th rib fragment adjacent to the descending thoracic aorta. On the following day, she developed worsening chest pain and a rapidly progressive left hemothorax with active contrast extravasation. Bleeding was initially suspected to arise from an intercostal artery, and transcatheter arterial embolization was attempted, but hemostasis was not achieved. Resuscitative endovascular balloon occlusion of the aorta was performed before interhospital transfer; however, she deteriorated to traumatic cardiac arrest during transport. An emergency left anterolateral thoracotomy in the emergency department achieved decompression and temporary hemorrhage control by evacuation of a large clotted hemothorax and intrathoracic gauze packing, allowing transfer to the operating room. Intraoperatively, a focal laceration of the posterior descending thoracic aorta was identified and repaired with pledgeted sutures, and concomitant intercostal arterial bleeding was cauterized. The patient underwent a tracheostomy on POD 10 and was transferred alive for rehabilitation on day 22.

**CONCLUSIONS:**

Posterior, medially displaced left rib fractures adjacent to the descending thoracic aorta represent a threatened aorta configuration and warrant particular attention, even when the initial hemothorax is minimal. In such situations, early contrast-enhanced reassessment, close monitoring, and multidisciplinary planning are warranted. If hemothorax rapidly enlarges or the clinical course worsens, clinicians should reconsider aortic injury rather than attributing the bleeding solely to an intercostal source, and timely escalation to definitive operative hemorrhage control may be lifesaving, even after traumatic cardiac arrest.

## Abbreviations

CPR
: cardiopulmonary resuscitation
ED
: emergency department
PEA
: pulseless electrical activity
REBOA
: resuscitative endovascular balloon occlusion of the aorta
ROSC
: return of spontaneous circulation
TAE
: transcatheter arterial embolization

## INTRODUCTION

Rib fractures are common after blunt chest trauma. However, descending thoracic aortic laceration directly caused by a rib fracture fragment is rare and can be rapidly fatal. Published evidence consists predominantly of case reports and small case series describing delayed deterioration hours to days after injury, often presenting as sudden massive hemothorax, refractory shock, or cardiac arrest despite an initially non-alarming clinical course or imaging findings.^1–6)^ In addition, cases have been reported in which a sharp, medially displaced posterior rib fragment lies in close proximity to the descending aorta and is considered to pose an imminent risk of aortic laceration or rupture, prompting urgent or prophylactic intervention.^7,8)^ Across these reports, a recurring pattern is medially displaced posterior or lower left rib fracture fragments in close proximity to the descending thoracic aorta, with aortic injury not clearly evident at initial presentation but later manifesting as a rapidly progressive hemothorax.^1–6)^

Initial evaluation may not clearly identify the bleeding source in rib fracture–associated hemothorax, and delayed presentation of aortic laceration has been reported after an initially benign course.^1–6)^ When hemodynamic collapse ensues, prompt surgical exposure may be required for definitive hemorrhage control.^9)^

## CASE PRESENTATION

### Patient information

**Fig. 1:**
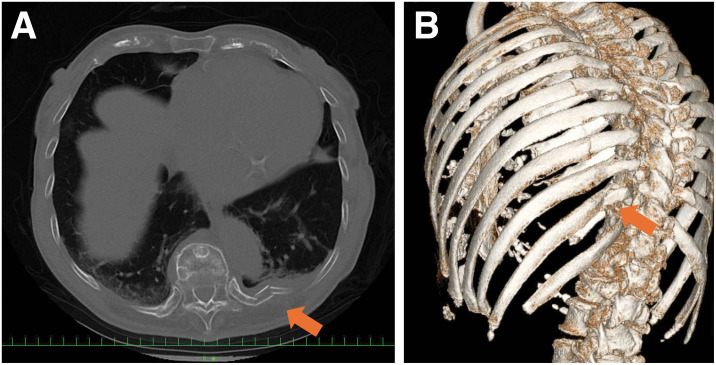
Initial CT at the referring hospital. (**A**) Non-contrast axial image demonstrating a medially displaced left posterior 11th rib fracture fragment adjacent to the descending thoracic aorta. (**B**) 3D VR reconstruction showing extensive bilateral rib fractures (left ribs 2–11 and right ribs 4–7), including the displaced posterior left lower rib fracture fragment in close spatial relationship to the descending thoracic aorta. The arrows indicate the medially displaced posterior left 11th rib fracture fragment. VR, volume-rendered

A 78-year-old woman fell and struck her chest against a tree, then presented to a local hospital, where extensive bilateral rib fractures were diagnosed (left ribs 2–11 and right ribs 4–7). The initial assessment reportedly showed minimal hemothorax, and she was managed conservatively. She had no other apparent traumatic injuries at presentation and was not taking antithrombotic medications. On initial imaging, a medially displaced left posterior 11th rib fracture fragment was adjacent to the descending thoracic aorta (**Fig. 1A**). 3D reconstruction demonstrated extensive bilateral rib fractures (**Fig. 1B**).

### Clinical findings and timeline

**Fig. 2:**
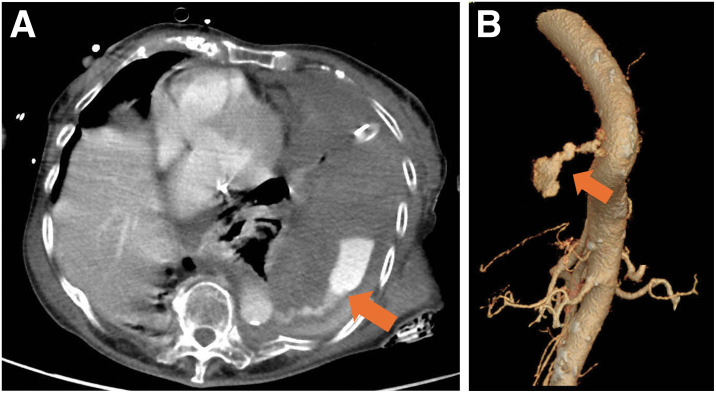
Contrast-enhanced CT after clinical deterioration. (**A**) Axial contrast-enhanced image demonstrating a massive left hemothorax with active contrast extravasation in the left posterior hemithorax. (**B**) 3D VR reconstruction demonstrating active contrast extravasation and its spatial relationship to the displaced posterior rib fractures. The arrows indicate active contrast extravasation. VR, volume-rendered

Approximately 40 hours after the initial presentation, the patient developed worsening chest pain and a rapidly progressive left hemothorax and returned to the local hospital. She was then transferred to the referring hospital, where the bleeding was initially attributed to an intercostal arterial injury. Contrast-enhanced CT demonstrated a massive left hemothorax with active contrast extravasation in the left posterior hemithorax (**Fig. 2A** and **2B**). TAE was attempted; however, hemostasis could not be achieved. As the hemorrhage remained uncontrolled after TAE, she was transferred to our hospital for an emergent thoracotomy and surgical hemostasis. Before transfer, REBOA was placed for temporary circulatory support at the previous hospital. During ambulance transport, she deteriorated to cardiac arrest. Upon arrival at our emergency department, she was in asystole, and CPR was ongoing. She arrived intubated with vascular access established; a left thoracic trocar catheter and a right femoral sheath were in place, and norepinephrine infusion had been initiated at the referring institution. Despite a transient ROSC, she developed recurrent PEA. Given profound shock with suspected obstructive physiology due to a massive intrathoracic clot burden, in addition to ongoing hemorrhage, an emergency left anterolateral thoracotomy was performed in the emergency room. A large amount of clotted hemothorax was evacuated, and temporary hemorrhage control was achieved with intrathoracic gauze packing. ROSC was obtained, and she was transferred to the operating room for definitive hemostasis.

### Diagnostic assessment

The initial chest CT at the first hospital was non-contrast and reportedly demonstrated multiple rib fractures (left ribs 2–11 and right ribs 4–7) with minimal hemothorax. A medially displaced left posterior rib fracture fragment (approximately at the 11th rib level) was noted to be in close proximity to the descending thoracic aorta. No other associated traumatic injuries were identified, and no major vascular injury was recognized on that initial non-contrast evaluation.

After clinical deterioration approximately 40 hours after the initial presentation, contrast-enhanced CT at re-presentation demonstrated active contrast extravasation in the left posterior hemithorax. The bleeding source was initially interpreted as intercostal arterial hemorrhage, and TAE was attempted without achieving hemostasis. Importantly, the initial CT showed minimal hemothorax and no obvious evidence of aortic injury, suggesting that the aortic lesion may have progressed and ruptured in a delayed fashion, possibly related to the sharp, medially displaced rib fragment adjacent to the aorta.

At the time of arrival at our hospital, the patient was in traumatic cardiac arrest. Definitive diagnosis was established intraoperatively, where a focal bleeding point on the posterior aspect of the descending thoracic aorta with surrounding localized mural irregularity was identified, along with hemorrhage from the opposing intercostal artery (10th–11th intercostal level).

### Therapeutic intervention

At the referring institution, TAE was attempted for presumed intercostal arterial bleeding, and REBOA was placed prior to transfer. However, because REBOA was inserted immediately before transport and the patient developed cardiac arrest en route, no interval imaging was available to verify balloon location or its relationship to the injured aortic segment. Upon arrival, the patient required advanced life support with repeated episodes of ROSC and recurrent cardiac arrest. Following temporary stabilization in the emergency department, definitive repair was undertaken in the operating room. During preparation, she again deteriorated to cardiac arrest, and open cardiac massage was performed through the thoracotomy incision while hemostatic procedures were initiated. The thoracotomy was extended, and the thoracic cavity was cleared of clot to identify the bleeding source. Manual compression of the bleeding point on the descending thoracic aorta resulted in immediate improvement in arterial pressure. The intercostal arterial bleeding was controlled by cauterization. The aortic laceration was repaired with pledgeted 3–0 polypropylene sutures.

### Follow-up and outcomes

Postoperatively, the patient was managed in the ICU and initially required full ventilatory support without spontaneous respiration. Because ventilator-associated pneumonia was suspected, antibiotics were administered, and a tracheostomy was performed on POD 10. She subsequently stabilized and was transferred alive to a secondary care facility for ongoing management and rehabilitation on POD 22.

## DISCUSSION

Delayed presentation of descending thoracic aortic injury secondary to rib fracture fragments is uncommon but has been repeatedly described in the literature largely as case reports and small case series. A consistent clinical theme is an initially non-alarming course, with minimal or absent hemothorax and no definitive evidence of great-vessel injury on early imaging, followed by abrupt deterioration hours to days later, most often as rapidly progressive massive hemothorax with refractory shock or cardiac arrest.^1–6)^ Our case mirrors this pattern: despite an initially minimal hemothorax, the patient developed catastrophic intrathoracic hemorrhage approximately 40 hours after the initial presentation and suffered traumatic cardiac arrest during interhospital transfer.

Two pathophysiologic mechanisms plausibly explain the delayed presentation. First, a sharply displaced posterior rib fragment may subsequently puncture the adjacent descending aorta as rib alignment changes with respiration, coughing, mobilization, or progressive displacement. In fact, prophylactic resection of the offending rib fragment has been reported when the rib stump was judged to threaten the aorta, even prior to frank bleeding.^7,8)^ Second, an initially limited aortic wall injury, such as a partial-thickness disruption or occult mural injury without immediate free rupture, may later rupture under persistent mechanical irritation or evolving wall failure. These mechanisms are not mutually exclusive and should be considered whenever posterior and/or lower left rib fractures are medially displaced toward the descending aorta.^1–6)^ In the present case, we could not determine definitively which mechanism predominated. However, when the patient re-presented approximately 40 hours after the initial presentation, active bleeding was already present on contrast-enhanced CT, although it was initially interpreted as intercostal arterial hemorrhage rather than aortic bleeding.

A major diagnostic pitfall is the attribution of hemothorax to intercostal arterial bleeding, which is common in rib fractures and can lead to anchoring. This diagnostic difficulty has also been emphasized in previous reports.^1–6)^ In our patient, the initial CT at the first hospital showed extensive rib fractures with minimal hemothorax, and no major vascular injury was recognized on the available initial non-contrast imaging; nevertheless, catastrophic hemothorax developed approximately 40 hours after the initial presentation, and a definitive diagnosis was established intraoperatively as a focal laceration of the descending thoracic aorta. Although TAE is an important treatment option for intercostal arterial bleeding associated with rib fractures, its limitations should be recognized when posterior rib fractures are medially displaced toward the descending thoracic aorta. In such situations, active extravasation in the posterior hemithorax, rapidly enlarging hemothorax, worsening symptoms, hemodynamic deterioration, or discordance between the presumed bleeding source and the clinical course should raise suspicion for aortic injury rather than isolated intercostal arterial bleeding. TAE may therefore be inappropriate or insufficient when the true source is aortic, and failure of TAE to achieve hemostatic control should prompt immediate reconsideration of the diagnosis and escalation to definitive operative hemorrhage control. Practically, we define a “threatened aorta configuration” as a sharp, medially displaced posterior rib fracture fragment directed toward, abutting, or in close proximity to the descending thoracic aorta, with potential for delayed aortic injury due to respiratory motion, positional change, or further displacement. When this configuration is present, clinicians should maintain a low threshold for contrast-enhanced CT or CT angiographic evaluation, close hemodynamic monitoring, and early multidisciplinary discussion, even if the initial hemothorax appears small.

Once hemodynamic collapse occurs, temporizing measures during transfer may be considered, but definitive hemorrhage control typically requires prompt operative exposure. Current hemothorax management guidelines emphasize timely drainage and escalation to operative management when ongoing bleeding or physiologic deterioration is present.^9)^ In the present case, the patient’s arrest during transport, repeated episodes of ROSC and recurrent PEA upon arrival, and profound shock with suspected obstructive physiology due to a massive clotted hemothorax, in addition to ongoing hemorrhage, supported immediate emergency department thoracotomy for decompression and damage-control hemorrhage control, including manual clot evacuation and intrathoracic packing, enabling transfer to the operating room for definitive repair. Thus, ED thoracotomy was not performed solely as a last-resort response to blunt traumatic arrest, but as a targeted intervention to relieve the obstructive intrathoracic clot burden and gain temporary hemorrhage control while bridging the patient to definitive aortic repair. Resuscitative thoracotomy algorithms from trauma societies similarly underscore the importance of rapid correction of immediately reversible causes of traumatic arrest and shock in appropriately selected patients.^10)^ Although details from the referring institution were limited, pre-transfer REBOA placement may have provided transient proximal control and a bridge during transport; contemporary practice guidelines describe REBOA as a potential adjunct for hemorrhagic shock in selected trauma and surgical patients when definitive control is pending.^11,12)^ However, in this case, REBOA was inserted immediately before departure from the referring hospital; the patient developed cardiac arrest during transport, and emergency thoracotomy was required immediately upon arrival. Therefore, no post-placement imaging was available, and we could not determine whether the expanded balloon was directly compressing the injured aortic segment or precisely assess its hemodynamic contribution. Our case therefore illustrates a pragmatic sequence for catastrophic thoracic hemorrhage with impending arrest: rapid temporization when available, immediate decompression and damage control in the emergency department when indicated, and definitive repair once operative resources are available.

In patients with rib fractures, particularly medially displaced posterior or lower left fractures near the descending thoracic aorta, delayed aortic injury should be considered when hemothorax enlarges or physiology worsens. Such fracture patterns may represent a threatened aorta configuration and should prompt early contrast-enhanced reassessment, close monitoring, and multidisciplinary discussion, even when the initial hemothorax is small. Early recognition of a threatened aorta configuration and readiness for definitive operative hemorrhage control may be lifesaving. If the clinical course worsens or TAE fails to achieve hemostatic control, clinicians should reconsider aortic injury rather than attributing the bleeding solely to an intercostal source. This case illustrates that rapid escalation to emergency thoracotomy and definitive aortic repair can be lifesaving even after traumatic cardiac arrest.

**Fig. 3:**
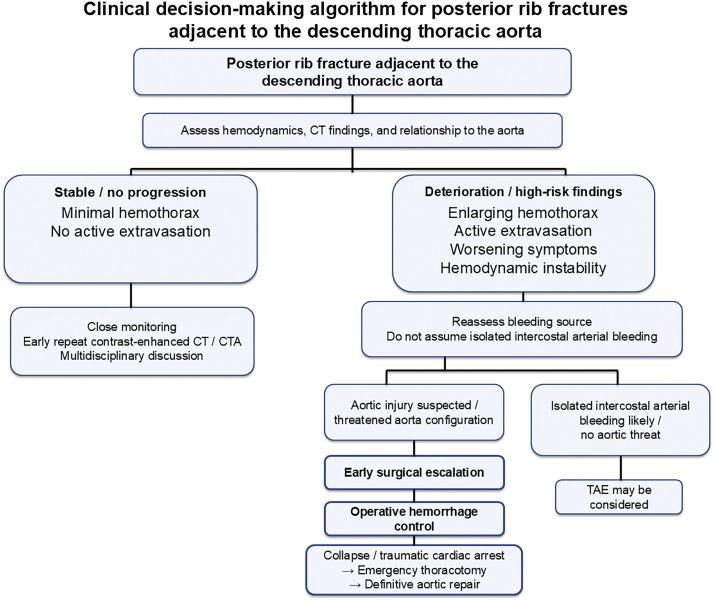
Schematic algorithm for clinical decision-making in posterior rib fractures adjacent to the descending thoracic aorta. The algorithm summarizes risk assessment and escalation of management, with emphasis on reassessment for possible aortic injury. TAE, transcatheter arterial embolization

From a practical standpoint, observation may be appropriate in selected stable patients, but a threatened aorta configuration should prompt a low threshold for contrast-enhanced reassessment and multidisciplinary discussion. Enlarging hemothorax, worsening symptoms, hemodynamic deterioration, or failure of TAE should trigger immediate reconsideration of aortic injury and escalation to operative hemorrhage control. A simple schematic algorithm summarizing this clinical decision-making process is shown in **Fig. 3**.

## CONCLUSIONS

Delayed presentation of descending thoracic aortic injury should be suspected in patients with posterior and medially displaced left rib fractures near the descending thoracic aorta, even when the initial hemothorax is minimal and early imaging shows no definite vascular injury. Such fracture patterns may represent a threatened aorta configuration and therefore warrant particular attention, including early contrast-enhanced reassessment and close monitoring. When hemothorax enlarges or hemodynamic status worsens, prompt reassessment and early multidisciplinary planning are essential. In cases of catastrophic deterioration, rapid escalation to emergency thoracotomy for decompression and damage-control hemorrhage control, followed by definitive aortic repair, may be lifesaving even after traumatic cardiac arrest.

## DECLARATIONS

### Availability of data and materials

The data are not publicly available because they contain information that could compromise patient privacy, but are available from the corresponding author upon reasonable request.
